# Inverted recruitment of autophagy proteins to the *Plasmodium berghei* parasitophorous vacuole membrane

**DOI:** 10.1371/journal.pone.0183797

**Published:** 2017-08-25

**Authors:** Jacqueline Schmuckli-Maurer, Vera Reber, Rahel Wacker, Annina Bindschedler, Anthony Zakher, Volker Theo Heussler

**Affiliations:** 1 Institute of Cell Biology, University of Berne, Berne, Switzerland; 2 Graduate School for Cellular and Biomedical Sciences, University of Berne, Berne, Switzerland; Institut national de la santé et de la recherche médicale - Institut Cochin, FRANCE

## Abstract

Selective autophagy and related mechanisms can act as variable defense mechanisms against pathogens and can therefore be considered as intracellular immune responses. When in hepatocytes, *Plasmodium* parasites reside in a parasitophorous vacuole (PV) and the PV membrane (PVM) is the main contact site between host cell and parasite. Early in infection, the PVM is directly labeled with host cell autophagy proteins LC3B and p62 (nucleoporin 62). We investigated the recruitment of different selective autophagy receptors and could show that mainly p62 and NBR1 (neighbour of BRCA1 gene 1) and to a lesser extent NDP52 (nuclear dot protein 52) associate with the PVM. To investigate the recruitment of these receptors to the PVM in *Plasmodium*-infected cells, we generated LC3B knock out HeLa cells. In these cell lines, autophagosome formation and autophagic flux are not different to those in WT cells. Unexpectedly, p62 and NBR1 recruitment to the PVM was strongly impaired in LC3B-negative host cells, suggesting that LC3B recruits both receptors to the PVM of *Plasmodium* parasites. We also noticed that LC3B recruited ubiquitin to the PVM. This indicates that, in comparison to classical selective autophagy, in *P*. *berghei*-infected cells the order of membrane labeling with autophagy proteins appears to be inverted from canonical ubiquitin-receptor-LC3B recruitment to LC3B-receptor and possibly ubiquitin.

## Introduction

Malaria is a devastating yet curable disease of global distribution, most prevalent in tropical and subtropical regions. According to the WHO report 2016, it remains one of the most deadly diseases in the world, with 200 million estimated cases and 400 thousand deaths per year. The causative agents of malaria are protozoan parasites of the genus *Plasmodium*.

When an infected female *Anopheles* mosquito takes a blood meal, it injects in the order of 100 *Plasmodium* sporozoites into the skin tissue [1]. From there sporozoites travel to the liver where they invade hepatocytes. When a sporozoite infects a liver cell, the host cell plasma membrane invaginates around the parasite, forming the parasitophorous vacuole membrane (PVM), in which liver stage schizogony takes place [2]. The PVM is the contact site between the parasite and its host. Despite its host cell origin, the PVM is quickly remodeled by the parasite and many *Plasmodium*-derived proteins can be found there [3,4]. The PVM has also been suggested as the target site for selective autophagy that occurs following sporozoite invasion [5].

In contrast to starvation-induced canonical autophagy, selective autophagy is nutrient insensitive and involves the selective degradation of proteins, organelles and pathogens. Ubiquitination is known to target proteins for degradation by the proteasome but can also label substrates for selective autophagy [6]. Such ubiquitin-conjugated or unfolded protein regions are recognised by a variety of autophagy receptors. They assemble through self-oligomerization and bind ubiquitin-like proteins (UBLs) of the LC3/GABARAP family. These UBLs are important for autophagosome formation, acting as a protein scaffold for the engagement of the autophagosome nucleation machinery and causing the expansion of the autophagosomal membrane [7–12]. The first selective autophagy receptor found in mammalian cells was nucleoporin 62 (p62), also called sequestosome 1 (SQSTM1) [13,14]. This protein is involved in the degradation of misfolded proteins, mitochondria, peroxisomes and pathogens [14–17]. Classically, p62 function depends on ubiquitination of the substrate. p62 contains an ubiquitin-binding domain (UBA), an LC3-interacting region (LIR) motif and a PB1 domain that mediates self-oligomerization. In selective autophagy, p62 is normally degraded together with its cargo. However, in autophagy-deficient cells, it is not degraded via macroautophagy and accumulates in the cytoplasm, therefore often being used to measure autophagic flux [11,18]. Another autophagy receptor is neighbor of *BRCA1* gene 1 (NBR1), whose domain organization resembles that of p62. NBR1 is an important receptor in degradation of peroxisomes (pexophagy) [19]. Optineurin (OPTN) can act as a receptor for misfolded proteins in both a ubiquitin-dependent and -independent manner [20]. OPTN has an UBA and a LIR motif and is also involved in xenophagy and mitophagy [21,22]. Nuclear dot protein 52 kDA (NDP52) can also act as an autophagy receptor in xenophagy. In *Salmonella* infection, NDP52 labeling of bacteria-containing vacuoles is dependent initially on galectin 8 and then on ubiquitin. [23].

Whereas autophagy-dependent selective elimination is a well-known host cell reaction against bacteria after invasion, there are only very few reports in the literature about selective autophagy in cells infected by eukaryotic parasites. Successful elimination by selective autophagy has been reported for the apicomplexan parasite *Toxoplasma gondii* [24,25]. However, it has also been shown that *T*. *gondii* is capable of actively evading this autophagic destruction by activating EGFR, which inhibits LC3 accumulation around the parasite [26]. More recently, we investigated selective autophagy events in *Plasmodium-*infected hepatocytes and showed that the PVM of *Plasmodium* liver stage parasites is rapidly and heavily labeled by the host cell-derived autophagy marker protein LC3B, indicating that the host cell quickly recognises the invader [5,27]. Interestingly, this labeling is greatly reduced in later stages of normally developing parasites, suggesting that the parasite is able to escape from this host cell response in order to successfully establish infection and undergo replication [5]. In contrast, persistent LC3B-labeling is linked to parasite growth arrest and to elimination, indicating that the host cell can defend itself successfully using autophagy or a related mechanism. Importantly, in addition to LC3B, ubiquitin and the autophagy receptor p62 also accumulate around the parasite [5]. However, the mechanisms that allow different autophagy marker proteins to be recruited to the PVM remained unknown. It was also unclear whether other autophagy receptors are involved in the observed selective labeling of the PVM and these questions are the basis of the work presented here.

We used the rodent parasite *Plasmodium berghei* to infect wild type and LC3B-deficient HeLa cells generated using CRISPR/Cas9 technology [28]. In contrast to what has been shown for classical selective autophagy, we found that p62, NBR1 and ubiquitin recruitment to the PVM depends on the presence of LC3B.

## Material and methods

### Cell culture, treatment and infection of HeLa cells

Wild type HeLa cells (a gift from Robert Menard, Pasteur Institute, Paris), LC3B^-/-^ and ATG5^-/-^ HeLa cells were cultured in Minimum Essential Medium with Earle’s salts (MEM EBS, 1-31F01-I, BioConcept, Allschwil, Switzerland), supplemented with 10% FCS (GE healthcare, Glattbrugg, Switzerland), 100 U/ml penicillin, 100 μg/ml streptomycin, and 2 mM L-glutamine (both BioConcept, BioConcept, Allschwil, Switzerland). Cells were cultured at 37°C and 5% CO_2_ and split using Accutase (Innovative Cell Technologies, San Diego, California, USA) diluted 1:1 in PBS.

For starvation, cells were rinsed 3 times with PBS and subsequently incubated in Earle’s Balanced Salt Solution (EBSS, E2888, Sigma-Aldrich, Buchs, Switzerland) for 2 h before fixation. For simultaneous treatment with chloroquine and rapamycin, cells were grown in MEM EBS supplemented with 10 μM chloroquine (C6628, Sigma-Aldrich, Buchs, Switzerland) and 250 ng/ml Rapamycin (R-500, LC Laboratories, New Boston, Massachusetts, USA) for 4 h before cell lysates were prepared.

For infection of HeLa cells, salivary glands of *P*. berghei-infected *Anopheles stephensi* mosquitoes were isolated and disrupted to release sporozoites. Sporozoites were incubated with cells in the smallest possible volume of MEM EBS medium containing 25 μg/ml Amphotericin B (4-05F00-H BioConcept, Allschwil, Switzerland) for 2h. Subsequently, they were rinsed and incubated in the respective medium containing 2.5 μg/ml Amphotericin B. Parasites used in this study have a *P*. *berghei* ANKA background. *Pb*mCherry parasites are phenotypically wild-type and show cytosolic localisation of the fluorescent mCherry protein [29].

### Transfection of HeLa cells

HeLa cells were harvested by Accutase treatment and 1 x 10^6^ cells were pelleted by centrifugation at 1000 x g. Cells were resuspended in Nucleofector V Solution (VVCA-1003, Lonza, Switzerland) and transfected with 1 μg of plasmid DNA using program T-028 of the Nucleofector 2b transfection device (Lonza, Switzerland) and according to the manufacturer’s instructions. For IFA analysis, cells were seeded onto glass cover slides in 24 well plates (#0117530, 13 mm No. 1.5, Marienfeld GmbH, Lauda-Königshofen, Germany)

### Plasmids

The various GFP expression vectors used are from Clontech. To generate the plasmid GFP-NBR1, the NBR1 cDNA was amplified by PCR using the forward primer CTGGTACCATGGAACCACAGGTTACTC and the reverse primer TGGGCCCTCAATAGCGTTGGCTGTA and plasmid pMXs-IP GFP-NBR1 (Addgene plasmid 38283, provided by Noboru Mizushima [30]) was used as a PCR template. The PCR product was subcloned into pJET1.2 (#K1232, Thermo Fisher Scientific, Reinach, Switzerland) via blunt end cloning and the NBR1 cDNA was verified by sequencing using the following primers: pJET1.2-fw CGACTCACTATAGGGAG; pJET1.2-rev ATCGATTTTCCATGGCAG; NBR1-915bp GCGAGCTGAGAAGAAACA. Finally the NBR1 cDNA was cloned into pEGFP-C1 using restriction enzymes KpnI and ApaI. To obtain the plasmid GFP-NDP52, the NDP52 cDNA was excised from plasmid pCR3-3x-Flag-NDP52 (a gift from Matias Faure, INSERM, Lyon) using the enzymes KpnI and ApaI. The NDP52-containing DNA fragment was cloned into pEGFP-C1 linearised with the same enzymes. For generating the GFP-OPTN plasmid, the OPTN cDNA was cut out from plasmid pOPTN-EGFP (Addgene plasmid 27052, provided by Beatrice Yue [31]) using the enzymes EcoRI and BamHI and ligated into pEGFP-C3 linearised with the same enzymes. The GFP-Gate16 plasmid is a gift from Zvulun Elazar, Weizmann Institute, Rehovot, Israel; pmRFP-ratLC3B (Addgene plasmid 21075, deposited by Tamotsu Yoshimori [32]), GFP-ubiquitin (Addgene plasmid 11928, deposited by Nico Dantuma [33]).

### Generation of an LC3B knockout cell line

The CRISPR/Cas9 nickase system described by [34] was used to knock out LC3B in HeLa cells. CRISPR guide RNA pairs (gRNAs) were designed to target exon 1 of the *LC3B* gene. Cloning of the 2 gRNA oligonucleotides, GATCCCTGCACCATGCCGT and GGCGACGACGCGAGGGTCC, into the plasmid pX335-U6-Chimeric_BB-CBh-hSpCas9n(D10A), (Addgene plasmid 42335, supplied by Feng Zhang [35]) was performed following the protocol of the Zhang laboratory [34]. HeLa cells were transfected with the two pX335 plasmids, each encoding one gRNA sequence and in addition with the plasmid pcDNA3.1(-)-Puro-EGFP-C1 (this plasmid is a gift from Erich Nigg, Biozentrum, University of Basel, Switzerland) following the transfection protocol described above. Transfected cells were seeded into 2 wells of a 6 well plate and 24 hours after transfection, 1 μg/ml puromycin was added for 48 h to select for transfected cells. After puromycin selection, cells were cultured for 10 days in MEM, passaged when confluent and subsequently plated into 10 96-well plates to obtain clonal cell lines. Single colonies were expanded and a first screen, to assess for the absence of LC3B, was carried out by immunofluorescence staining. Cells of the clonal cell lines were seeded into glass-bottom 96-well plates and grown over night. The next day, cells were starved by incubation in EBSS for 2h to induce formation of autophagosomes, which can be visualised by fluorescence microscopy. Cells were then fixed and stained with α-LC3B antibodies. Putative positive clones that lacked an LC3B staining were further analysed by immunoblotting. Clones that showed no LC3B signal by Western blot analysis were further analyzed on the genomic DNA level. Genomic DNA was isolated from these cells using the QuickExtract^™^ DNA extraction solution 1.0 (Epicentre, Madison, Wisconsin, USA) and the regions of interest were amplified using PCR (PCR primers are listed in S1 Table). PCR fragments were cloned into pGEM-T-Easy (#1360, Promega, Madison, Wisconsin, USA) and at least 20 plasmids of each cell line were analyzed by sequencing using standard primers (T7: TAATACGACTCACTATAGG; SP6: ATTTAGGTGACACTATAG). Sequencing of the targeted genomic regions of knockout lines confirmed the presence of DNA alterations that lead to the knockout phenotype. Sequences of the different primers used for analyzing the three LC3B knockout cell lines and the detailed results of this analysis are summarised in S1 Table.

### Protein lysates and western blotting

Cells were seeded into 24- or 6-well plates to reach confluency the next day. 24h later, after the appropriate treatment, cells in each well were rinsed with PBS and lysed for 30 min on ice with 50 μl or 200 μl ice-cold RIPA buffer (50mM Tris-HCl pH 7.0, 1% NP-40, 0.5% Na-deoxicholate, 150mM NaCl, 2mM Na-Fluoride, 0.1% SDS, Complete^™^ Mini EDTA-free protease inhibitor cocktail (Sigma-Aldrich, Buchs, Switzerland)) and incubated on ice for 30 min with occasional shaking. Next, the lysate was centrifuged at 16,000 rcf / 15 min / 4°C. The supernatant was transferred into a fresh tube and mixed with Laemmli sample buffer. Proteins were denatured by incubating at 90°C for 5 min and then separated on 12% or 15% (for LC3B) SDS PAGE gels, followed by transfer to nitrocellulose membrane. 5% milk in TBST was used to block the membranes and for incubation with rabbit anti-LC3B (L7543, 1:1500, Sigma-Aldrich, Buchs, Switzerland), mouse anti-p62 (M162-3, 1:1000, MBL International, Woburn, Massachussetts, USA), chicken anti-GAPDH (AB2302, 1:5000, EMD Millipore, Darmstadt, Germany) and mouse anti-alpha-Tubulin (T9026, 1:1000, Sigma-Aldrich, Buchs, Switzerland). For secondary antibody incubation, anti-rabbit, anti-mouse IgG 800 CW IRDye and anti-chicken and anti-mouse IgG 680 LT IRDye (Li-Cor Biosciences, Lincoln, Nebraska, both 1:10,000) were diluted in 5% milk in TBST. A Li-Cor Odyssey Imaging system (Li-Cor Biosciences, Lincoln, Nebraska) was used for detection.

### Indirect immunofluorescence analysis

For IFA analysis, cells were grown on glass cover slips (#0117530, Marienfeld GmbH, Lauda-Königshofen, Germany). After indicated time periods, cells were fixed with 4% paraformaldehyde in phosphate-buffered saline (PBS; 137 mM NaCl, 2.7 mM KCl, 10 mM Na_2_HPO_4_, 1.8 mM KH_2_PO_4_, pH 7.4) for 10 min (all incubations at room temperature). Permeabilization was performed for five minutes in 0.1% Triton X-100 (T8787, Sigma-Aldrich, Buchs, Switzerland) in PBS or in 10 mg/ml digitonin (D141, Sigma-Aldrich, Buchs, Switzerland) in PBS for the anti-LC3B antibody. After washing with PBS, unspecific binding sites were blocked by incubation in 10% FCS/PBS for 10 min followed by incubation with primary antibody in 10% FCS-PBS for at least 1 hour. Primary antibodies used were mouse anti-LC3B (M152-3, 1:500, MBL International, Woburn, Massachusetts, USA), rabbit anti-UIS4 antiserum (provided by P. Sinnis, Baltimore, USA, 1:500), rabbit anti-GFP (SP3005P, 1:1000, Acris, Herford, Germany), mouse anti-GFP (11814460001, 1:1000, Roche Life Science, Rotkreuz, Switzerland), mouse anti-p62 (M162-3, 1:500, MBL International, Woburn, Massachusetts, USA), monoclonal rabbit anti-NBR1 (D2E6) (#9891, 1:1000, Cell Signaling Technology, Danvers, Massachusetts, USA), monoclonal mouse anti-Ubiquitin (FK2) (PW8810, 1:1000, Enzo Life Sciences Farmingdale, New York). Subsequently, cells were incubated with fluorescently labeled secondary antibodies in 10% FCS/PBS for at least 45 min using the following antibodies: anti-mouse Alexa488 (A11001, 1:1000, Invitrogen, Carlsbad, California, USA), anti-rabbit Alexa488 (A11008, 1:1000, Invitrogen, Carlsbad, California, USA), anti-mouse Alexa594 (A11032, 1:1000, Invitrogen, Carlsbad, California, USA), anti-rabbit Cy5 (Dianova, 1:1000, Hamburg, Deutschland) and anti-mouse Cy5 (Dianova, 1:1000, Hamburg, Deutschland). DNA was visualised by staining with 1 μg/ml DAPI (Sigma-Aldrich, Buchs, Switzerland) in PBS for 5 min. Cells were mounted on microscope slides with ProLong^®^ Gold Antifade Mountant (P36930, Thermo Fisher Scientific, Reinach, Switzerland) and analysed using a Leica TCS SP8 confocal microscope. For quantification of autophagy markers, a widefield Leica DM5500B epifluorescence microscope was used. Image processing was performed using Fiji software.

### Quantification of autophagy markers

#### Association of autophagy receptors with *P*. *berghei* parasites

HeLa WT cells were transfected with plasmids expressing GFP-tagged versions of the autophagy receptors NBR1, NDP52, OPTN. Approximately 24 hours after transfection, cells were infected with *Pb*mCherry parasites and 6 hours post-infection, cells were fixed and stained with antibodies and monitored by fluorescence microscopy. For p62, non-transfected cells were infected and endogenous p62 was stained with antibodies. Receptor association with the *P*. *berghei* PVM was quantified visually. The slide was screened in the red channel for mCherry-expressing parasites. If the parasite PVM could also be clearly detected in the green channel (i.e. labeled with one of the autophagy receptors), this parasite was considered as positive. 60–100 parasites were counted for each group in each of two individual experiments.

#### Quantification of p62 association with *P*. *berghei*

HeLa WT and LC3B knockout cells were infected with *Pb*mCherry and 6 hours post-infection were fixed and stained with anti-p62 antibodies. Quantification was performed visually by counting the parasites that were stained strongly or weakly by the antibody. If the signal covered more than 30% of the area surrounding the parasite the signal was considered as strong. If less than 30% of the surrounding area was covered with p62, the signal was counted as weak or as having no association. Between 100 and 130 parasites were analyzed in each of two individual experiments.

For quantification of p62 association in the add-back experiment, HeLa WT and HeLa LC3B knockout cell lines were transfected with pmRFP-LC3B. Approximately 24 hours after transfection, cells were infected with *Pb*mCherry and 6 hours post-infection were fixed and stained with anti-p62 antibodies. Cells that were RFP-LC3B-positive and infected with *Pb*mCherry were monitored for p62 localization. Quantification was performed visually as described above. 60–120 parasites were analyzed in each of two individual experiments.

For calculating the Pearson’s correlation coefficient in the add-back experiment, cells were transfected and infected as described in the paragraph above. To visualise p62, fixed cells were stained with a mouse monoclonal anti-p62 antibody followed by a secondary anti-mouse antibody labeled with Alexa488. To visualise RFP-LC3B, we used a polyclonal rabbit anti-GFP (Acris) antiserum, which also stains RFP (but not mCherry) followed by a secondary anti-rabbit antibody labeled with Cy5. Confocal pictures of the green and far-red channels were taken and a Pearson’s correlation coefficient was calculated using the Coloc2 tool of the Fiji software.

#### Quantification of GFP-NBR1 association with *P*. *berghei*

HeLa WT and LC3B knockout cells were transfected with GFP-NBR1 and approximately 18 hours after transfection were infected with *Pb*mCherry. Cells were fixed 6 hours post-infection and stained with anti-NBR1 antibodies. Quantification was performed visually by counting the parasites that were stained strongly or weakly by the antibody. If the signal covered more than 30% of the area surrounding the parasite the signal was considered as strong. If less than 30% of the surrounding area was covered with GFP-NBR1 the signal was counted as weak. Between 84 and 111 parasites were analyzed in each of two individual experiments.

For quantification of GFP-NBR1 association in the add-back experiment, HeLa WT and HeLa LC3B knockout cell lines were transfected with pmRFP-LC3B and GFP-NBR1. Approximately 24 hours after transfection, cells were infected with *Pb*mCherry and 6 hours post-infection cells were fixed and stained with monoclonal anti-NBR1 and anti-LC3B antibodies. Cells that were RFP-LC3B-positive and GFP-NBR1-positive and infected with *Pb*mCherry were monitored for GFP-NBR1 localization. Quantification was performed visually as described above. 50 parasites were analyzed in each of two individual experiments.

For calculating the Pearson’s correlation coefficient in the add-back experiment, HeLa WT and HeLa LC3B knock out cell lines were transfected with pmRFP-LC3B and GFP-NBR1. Approximately 24 hours after transfection, cells were infected with WT *P*. *berghei* sporozoites and 6 hours post-infection, cells were fixed and stained with anti-NBR1 and anti-LC3B antibodies. Cells that were RFP-LC3B-positive and GFP-NBR1-positive and infected with *P*. *berghei* were analysed by confocal microscopy. Pearson’s correlation coefficient was calculated using Coloc2 module of the Fiji software.

#### Quantification of ubiquitin association with *P*. *berghei*

HeLa WT and LC3B knockout cells were treated as described for the p62 quantification experiments. Fixed cells were stained with anti-ubiquitin antibodies. Quantification was performed in the same way as for p62. Between 77 and 103 parasites were analyzed in each of two individual experiments.

For quantification of the ubiquitin association in the add-back experiment, cells were treated as described for p62 quantification. Fixed cells were stained with anti-ubiquitin antibodies. Quantification was performed in the same way as described for p62. Between 53 and 83 parasites were analyzed in each of two individual experiments.

For calculating the Pearson’s correlation coefficient in the add-back experiment, HeLa WT and HeLa LC3B knock out cell lines were transfected with pmRFP-LC3B. Approximately 24 hours after transfection, cells were infected with WT *P*. *berghei* sporozoites and 6 hours post-infection cells were fixed and stained with anti-ubiquitin and anti-LC3B monoclonal antibodies. Cells that were RFP-LC3B-positive and GFP-NBR1-positive and infected with *P*. *berghei* were analyzed by confocal microscopy. Pearson’s correlation coefficient was calculated using Coloc2 module of the Fiji software.

### Statistical analysis

Two groups were compared using a 2-tailed, unpaired Student t-test. All statistical analyses were carried out using Prism 4.0c for Mac, GraphPad Software, San Diego, California, USA. P values of less than 0.05 were considered to indicate statistical significance. Pearson’s correlation coefficients were calculated with the Coloc 2 tool in Fiji by defining a region of interest around the *P*. *berghei* parasite.

## Results

### p62 and NBR1 are recruited to the PVM in *Plasmodium*-infected HeLa cells

**Fig 1:**
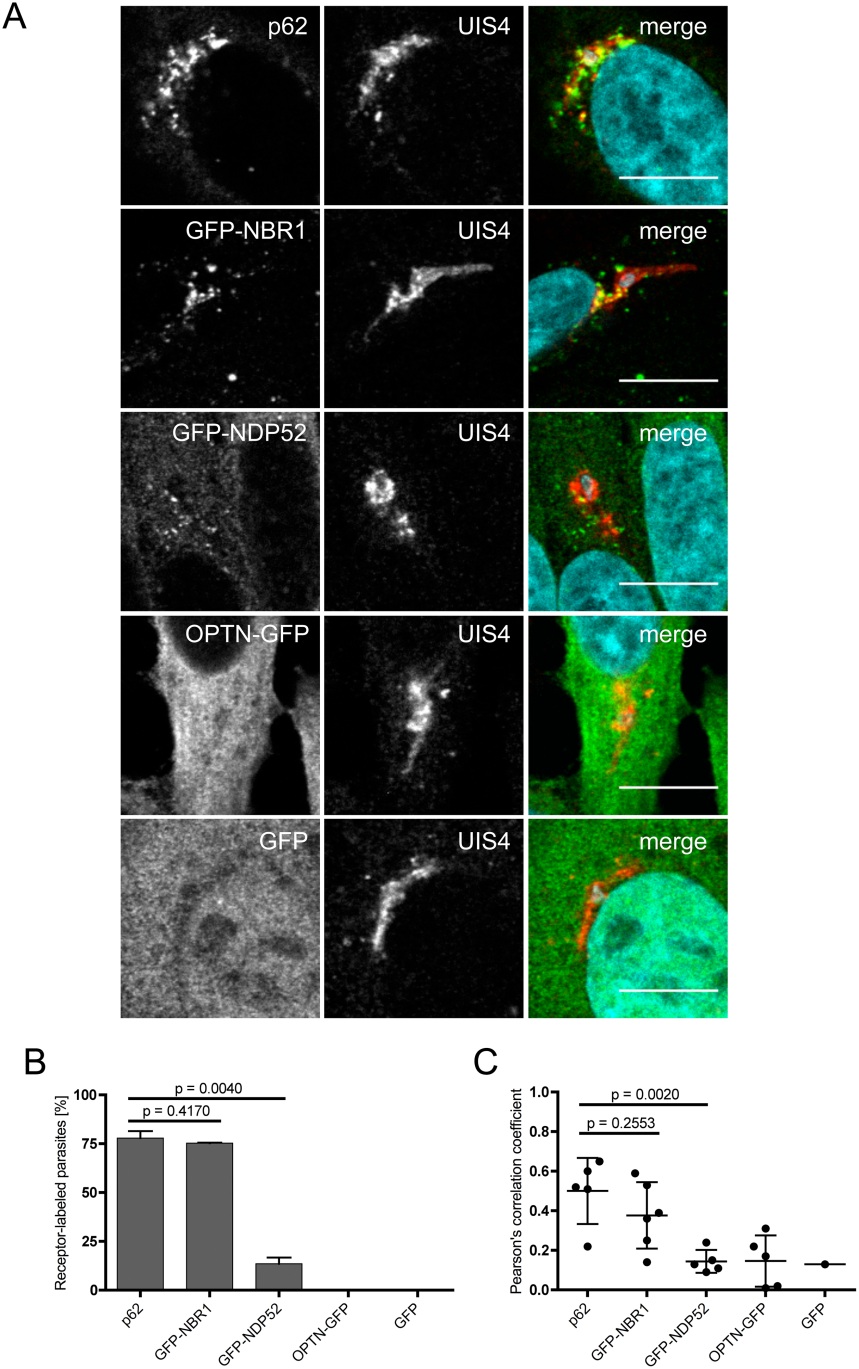
Autophagy receptors p62 and NBR1 localise to the parasitophorous vacuole membrane of *Plasmodium berghei*. **(A)** HeLa cells were infected with *Plasmodium berghei*. The parasitophorous vacuole membrane (PVM) of the parasite was stained with antibodies (UIS4, red). To visualise the autophagy receptors, cells were stained with antibodies (p62, green) or transfected 24 hours before infection with plasmids expressing GFP fusion proteins (GFP-NBR1, GFP-NDP52, OPTN-GFP, GFP, all shown in green). Infected cells were fixed 6 hours post-infection, stained with antibodies and analysed by confocal microscopy. DNA was stained with DAPI. Scale bar 10 μm **(B)** Numbers of receptor-labeled *P*. *berghei* parasites were determined by fluorescence microscopy. 60–100 parasites were counted in two separate experiments. Numbers of labeled parasites are expressed as percentages, error bars show standard deviations, p values were calculated using a t-test. **(C)** Pearson's correlation coefficients were calculated from at least 5 images, except for cells expressing GFP only. Standard deviations are depicted, p values were calculated using a t-test.

When liver cells are infected with *P*. *berghei*, the parasites are immediately recognised and the PVM is labeled with the autophagy marker proteins LC3B, ubiquitin and p62 [5]. Approximately half of the invaded parasites are eliminated early in infection. Based on the PVM labeling, selective autophagy is obviously a possible means of elimination. The observed mechanism appears, however, to be different from classical selective autophagy, which is characterised by the generation of an additional membrane around the invading pathogen. To further analyze the selective PVM labeling we infected HeLa cells expressing GFP-tagged selective autophagy receptors, NBR1, NDP52 and OPTN in *P*. *berghei*-infected cells. Cells were then fixed 6 hours post-infection and stained with α-GFP or α-p62 antibodies to localize the different receptors and additionally with anti-UIS4 antibodies to allow visualization of the PVM. The localization of the different receptors was then analyzed by confocal microscopy. Fig 1 shows that endogenous p62 and GFP-NBR1 clearly associate with the PVM of the parasite. GFP-NDP52 shows some PVM localization but is primarily in the cytoplasm and OPTN-GFP has an entirely cytoplasmic localization. The control protein, GFP, localises to both the cytoplasm and nucleus. A quantitative assessment of this localization experiment (Fig 1B and 1C) revealed that in the majority of infected cells, the PVM was clearly labeled with p62 (77.9%) and with GFP-NBR1 (75.3%). In contrast, GFP-NDP52 association with the PVM was only found in 13.5% of infected cells and OPTN-GFP and GFP did not localise to the PVM in infected host cells.

### Generation of LC3B knock out cell lines

**Fig 2:**
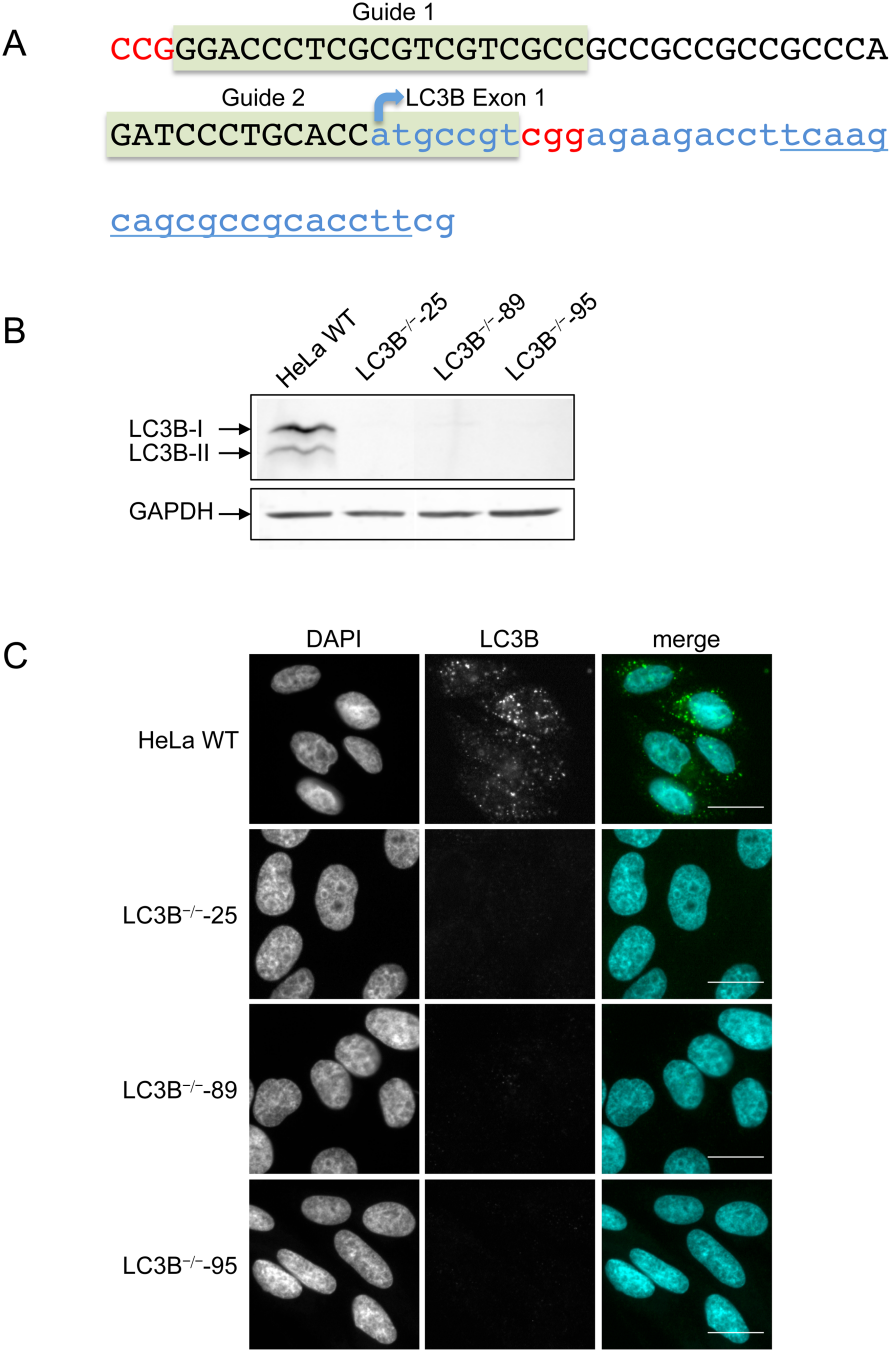
Generation of LC3B knockout HeLa cell lines. **(A)** Genomic region of the *LC3B* gene. Exon 1 is shown in lowercase blue, the 5’-upstream region is printed in black capitals. Binding regions of the two gRNAs are highlighted in green and PAM sequences are shown in red. **(B)** Western blot of HeLa WT and three LC3B knock out cell lines to confirm the lack of LC3B protein in three clonal cell lines. GAPDH was used as a loading control. **(C)** IFA analysis of HeLa WT and three LC3B knock out cell lines. Cells were starved for 2 hours in EBSS, fixed, stained with anti-LC3B antibodies (green) and analysed by fluorescence microscopy. DNA was visualised with DAPI (blue). Scale bar 20 μm.

Having confirmed that several selective autophagy receptors are recruited to the PVM, we next explored the mechanism of how this occurs. It is well known that LC3 family proteins interact with autophagy receptors through their LIR domains [11]. To further analyze this process on the PVM, we constructed an LC3B knock out cell line in HeLa cells using CRISPR/Cas9 genome editing. To reduce potential off-target effects we used a double nick strategy [34] with two guide RNA (gRNA) sequences targeting the first exon of *LC3B* (Fig 2A). Introducing staggered nicks leads to a DNA double strand break that will be imperfectly repaired by the non-homologous end joining repair pathway, resulting in insertion/deletion mutations that will ultimately disrupt the gene function. The plasmids encoding the gRNAs and the Cas9 nickase were co-transfected together with a plasmid harboring a puromycin resistance cassette. Puromycin-selected and expanded clones were first tested by immuno fluorescence analysis using α-LC3B antibodies and then by immunoblot analysis. Three individual clones were found to be LC3B-deficient (Fig 2B and 2C). From these three clones the genomic DNA region was amplified by PCR, the PCR fragments were subcloned and at least 20 plasmids of each clone were sequenced. All three clones showed modifications at the predicted locus (see S1 Table). For simplification, we present in the subsequent main figures the results from clone 25 only. The corresponding figures for clones 89 and 95 are available as supplementary data.

### LC3B knockout does not block canonical autophagy

**Fig 3:**
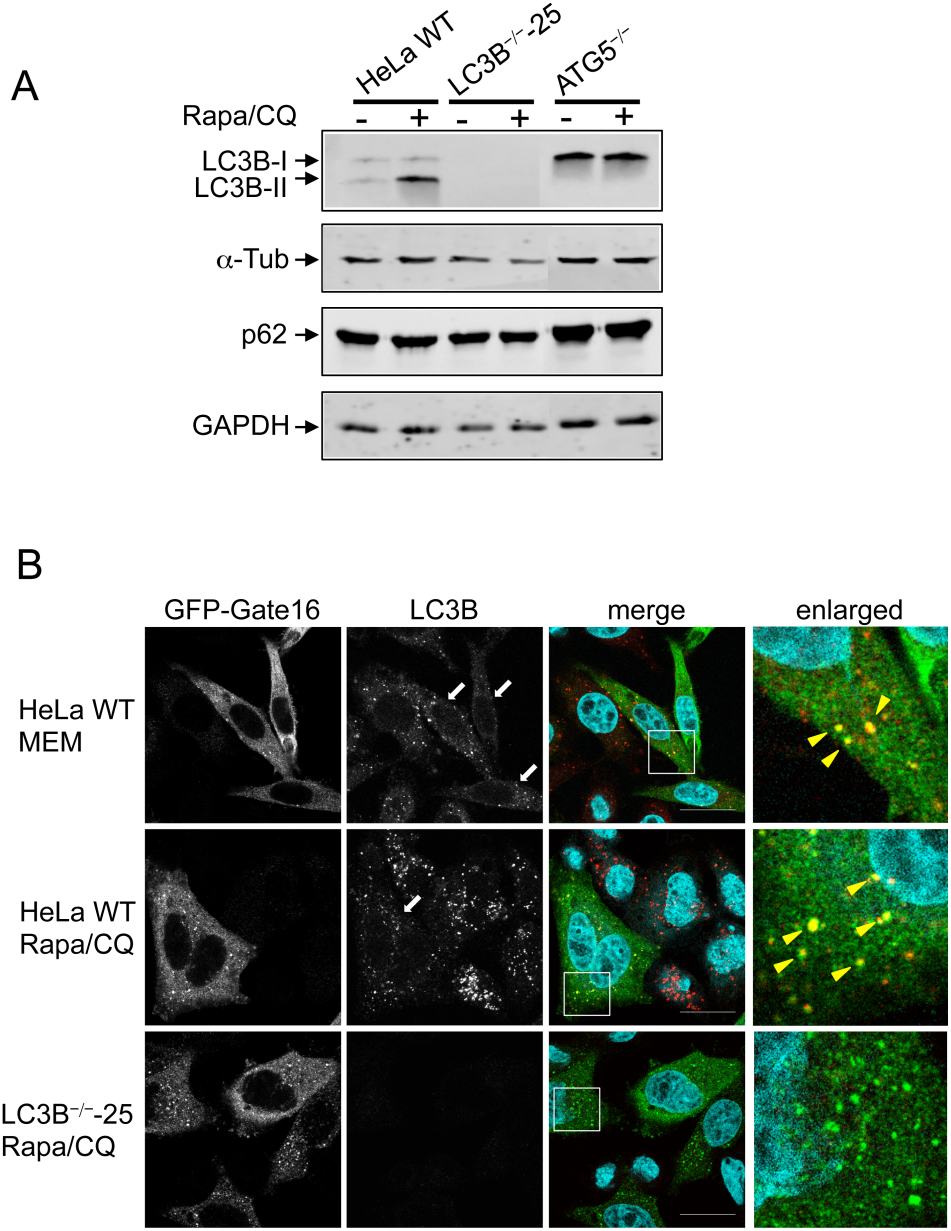
LC3B knockout cells are able to undergo canonical autophagy. **(A)** Representative western blot of non-infected HeLa WT, ATG5-knockout cells and one clonal LC3B-knockout cell line left untreated or treated simultaneously with 10 μM chloroquine and 250 ng/ml rapamycin for 4 hours. **(B)** HeLa WT and HeLa LC3B knockout cells ectopically expressing GFP-Gate16 were left untreated or treated with 10 μM chloroquine and 250 ng/ml rapamycin for 4 hours. Fixed cells were stained with anti-GFP antibodies to visualise Gate16 (green) or anti-LC3B antibodies (red). DNA was stained with DAPI (blue). White arrows in the LC3B panel indicate Gate16-transfected cells. Yellow arrowheads in the enlarged pictures indicate autophagic structures where Gate16 and LC3B colocalise. Scale bar 20 μm. Experiments using other clonal LC3B-knockout cells are shown in S1B Fig.

Before we started infection experiments with the newly generated LC3B-deficient clones, we wanted to know whether they are still proficient in executing canonical autophagy, including formation of autophagosomes and fusion with lysosomes. We therefore treated WT and LC3B^-/-^ HeLa cells with rapamycin and chloroquine. Rapamycin is an inhibitor of mTOR and activates autophagy. Chloroquine inhibits the fusion of autophagosomes with lysosomes and therefore the autophagic flux. Proteins that are located in the autophagosomes, such as LC3B-II and p62, are thus expected to accumulate [36]. In WT HeLa cells, LC3B-II indeed accumulated after rapamycin and chloroquine treatment, confirming the inhibition of LC3B turnover (Fig 3A and S1A Fig), although no significant accumulation of p62 was observed. As a solid control for the inhibition of the autophagic flux, we used HeLa cells lacking the ATG5 protein [37]. These cells are not able to generate LC3B-II and show a clear accumulation of p62 even without rapamycin and chloroquine treatment. The short drug treatment of ATG5-deficient cells shows no additional effect because autophagy is always blocked in this cell line (Fig 3A and S1A Fig). For the LC3B-deficient cell lines, no difference could be detected in respect to the autophagic flux, suggesting that LC3B is not required for autophagy in HeLa cells. To confirm this result, we also investigated the formation of autophagosomes in WT and LC3B knockout cell lines using Gate16, another typical autophagosome marker protein, belonging to the LC3/GABARAP family of proteins. Gate16 is, similarly to LC3B, lipidated and incorporated into the autophagosomal membrane [38]. The different cell lines were transfected with a plasmid encoding GFP-Gate16 and again treated with rapamycin and chloroquine. To visualise autophagosomes, cells were fixed and stained with anti-GFP and anti-LC3B antibodies. In WT HeLa cells, GFP-Gate16-positive vesicles co-localise with LC3B-positive vesicles, confirming that they are true autophagosomes (Fig 3B and S1B Fig, second top panel). Interestingly, LC3B-deficient HeLa cells are still fully proficient in forming autophagosomes, which are represented by GFP-Gate16 positive foci (Fig 3B and S1B Fig, 3 lower panels) confirming the immunoblot analysis (Fig 3A and S1A Fig). The observation that LC3B-deficient cells are still fully capable of undergoing autophagy was expected since Nguyen and coworkers have recently shown that cells deficient in all ATG8 family proteins are still able to form autophagosomes [39].

### LC3B recruits p62 to the PVM in *P*. *berghei*-infected cells

**Fig 4:**
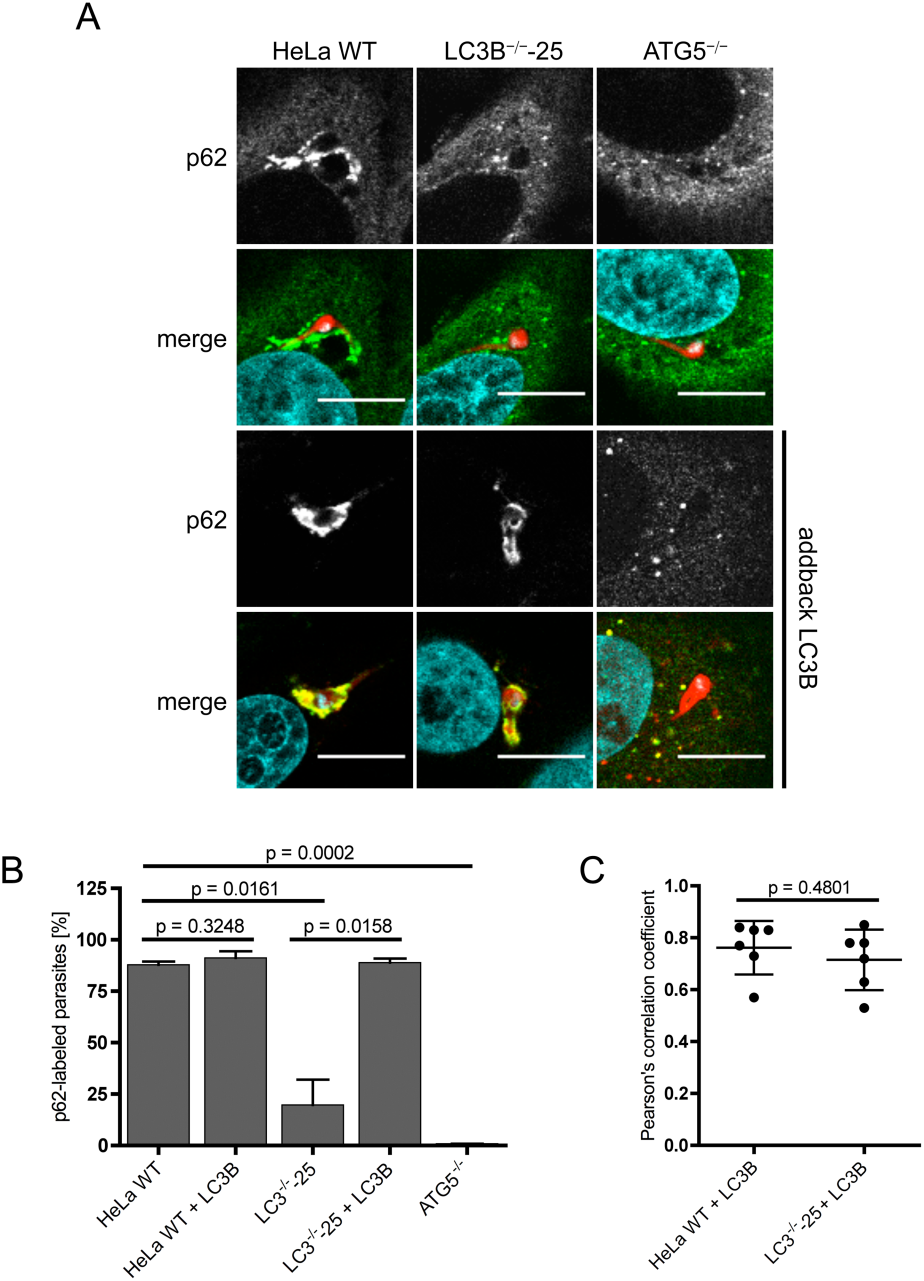
LC3B recruits p62 to the PVM. **(A)** HeLa WT, HeLa LC3B- and ATG5-knockout cells were infected with *Pb*mCherry (red). 6 hours post-infection, cells were fixed and stained with α-p62 antibodies (green). All cell lines were transfected with RFP-LC3B (two lower panels) and infected 17 hours after transfection with *P*. *berghei* sporozoites expressing mCherry (red). p62 (green) was visualised using a specific monoclonal α-p62 antibody. DNA was labeled with DAPI (blue). Cells were analyzed by confocal microscopy. Scale bar 10 μm. **(B)** Numbers of p62-labeled *P*. *berghei* parasites in non-transfected and in RFP-LC3B-transfected cells were determined by fluorescence microscopy. 100–130 parasites were analyzed in non-transfected HeLa cells and 60–120 parasites were analyzed for RFP-LC3B-transfected HeLa cells. Two individual experiments were carried out. Labeled parasites are expressed as percentages. Standard deviations are depicted. **(C)** Pearson’s correlation coefficient of p62 and RFP-LC3B were calculated for six individual *P*. *berghei* parasites in HeLa WT and LC3B^-/-^ cells transfected with RFP-LC3B. The mean values of 0.762 (HeLa WT) and 0.715 (LC3B^-/-^) indicate a strong co-localization of p62 and RFP-LC3B. Depicted are standard deviations.

To learn more about the kinetics of LC3B and p62 labeling of the PVM, we next infected HeLa cells lacking a functional LC3B gene and visualised p62 with anti-p62 antibodies 6 hours post infection. According to the existing literature on selective autophagy [11,40], we expected that the PVM would first be ubiquitinated, which would in turn attract p62 binding. The ubiquitin-bound p62 is then expected to recruit LC3B via a LIR domain. Contrary to this expectation, we found that in infected LC3B-deficient cells, p62 labeling of the PVM was strongly reduced compared to in parasite-infected WT HeLa cells (Fig 4A and 4B and S2A and S2B Fig). This implies that in *P*. *berghei*-infected cells, p62 association with the PVM mainly depends on LC3B. To confirm this observation, we also infected ATG5^-/-^ cells and monitored p62 localization. In infected ATG5^-/-^ cells, LC3B cannot be lipidated and is thus not integrated into the PVM [5]. In support of the previous result, p62 did not significantly associate with the PVM in ATG5^-/-^ cells. In a very small number of infected cells, some dot-like structures could be seen close to the parasite (Fig 4A and 4B and S2A and S2B Fig).

To finally prove that the observed lack of p62 recruitment to the PVM was indeed due to the lack of LC3B and was not an unspecific artifact of cell transfection and cloning, we transiently complemented all three LC3B-deficient HeLa cell lines with a plasmid encoding an RFP-LC3B fusion protein and infected them with *P*. *berghei* sporozoites. In all three RFP-LC3B-complemented LC3B^-/-^ cell lines, the p62 localization to the PVM was comparable to in WT HeLa cells (Fig 4A and S2A Fig, two lowest panels). In all three complemented LC3B^-/-^ cell lines, more than 85% of the parasites showed a strong association with p62 (Fig 4B and S2B Fig). For one LC3B knock out cell line complemented with RFP-LC3B, the co-localization coefficient was calculated, confirming a strong association of RFP-LC3B and p62 at the PVM (Fig 4C). Indeed, in all RFP-LC3B-transfected cells harboring a parasite, we observed a very strong PVM labeling with p62, suggesting that indeed LC3B is responsible for the recruitment of p62 and not the other way around. Together, these results provide strong evidence that LC3B plays a decisive role in recruiting the autophagy receptor p62 to the PVM in *P*. *berghei*-infected host cells.

### LC3B recruits GFP-NBR1 to the PVM in *P*. *berghei*-infected cells

**Fig 5:**
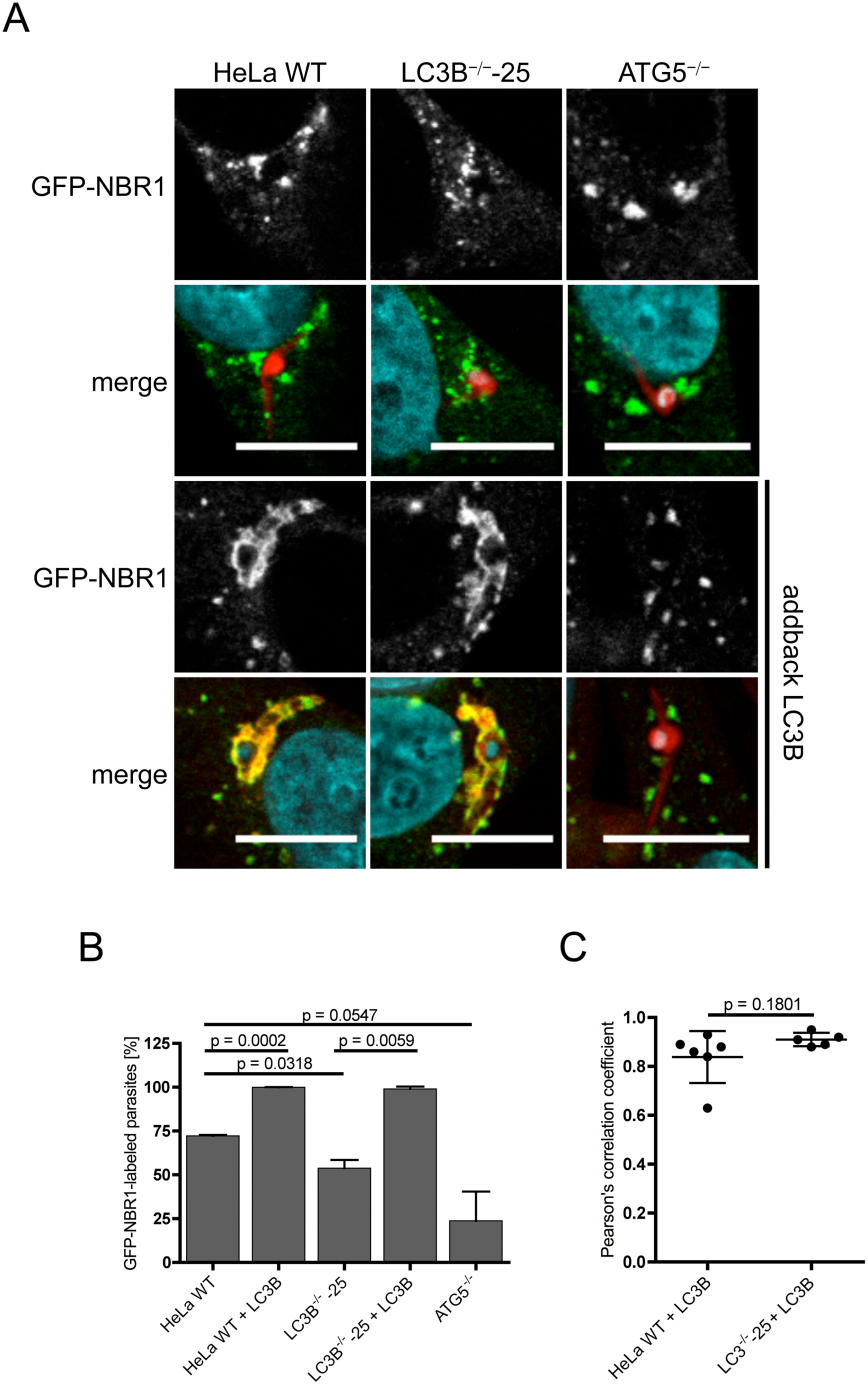
LC3B recruits GFP-NBR1 to the PVM. **(A)** HeLa WT, HeLa LC3B- and ATG5-knockout cells were infected with *Pb*mCherry (red). 6 hours post-infection, cells were fixed and stained with anti-NBR1 antibodies (green) as a control. Control and knockout cell lines were transfected with RFP-LC3B and GFP-NBR1 expression constructs (two lower panels) and infected 17 hours after transfection with *Pb*mCherry sporozoites (red). RFP-LC3B (red) and GFP-NBR1 (green) were visualised using mouse monoclonal anti-LC3B and rabbit monoclonal anti-NBR1 antibodies. DNA was labeled with DAPI (blue). Cells were analyzed by confocal microscopy. Scale bar 10 μm. **(B)** Numbers of GFP-NBR1-labeled *P*. *berghei* parasites in non-transfected and in RFP-LC3B-transfected cells were determined by fluorescence microscopy. 84–111 parasites were analysed in the non-transfected HeLa cells and 50 parasites were analysed for the RFP-LC3B-transfected HeLa cells. Two individual experiments were carried out. Labeled parasites are expressed as percentages. Standard deviations are depicted. **(C)** Pearson’s correlation coefficient of GFP-NBR1 and RFP-LC3B was calculated from at least five individual *P*. *berghei* parasites in HeLa WT and LC3B^-/-^ cells transfected with RFP-LC3B. The mean values of 0.838 (HeLa WT) and 0.910 (LC3B^-/-^complemented with RFP-LC3) indicate a strong co-localization of p62 and RFP-LC3B. Depicted are standard deviations.

As p62 and NBR1 are described as interacting with each other [41] and as we observed NBR1 at the PVM of *P*. *berghei-*infected cells (Fig 1), we next analyzed NBR1 localization in LC3B- and ATG5-deficient cell lines. NBR1 was ectopically expressed as a GFP-NBR1 fusion protein. HeLa WT, LC3B^-/-^ and ATG5^-/-^ cells were transfected with GFP-NBR1 and infected with *Pb*mCherry. Six hours post-infection, cells were fixed, stained with antibodies and GFP-NBR1 localization was qualitatively and quantitatively evaluated by microscopy. GFP-NBR1 clearly localised to the PVM of infected HeLa WT (72%) cells and to some extent also to the PVMs of parasite-infected LC3B^-/-^ cells (53%) and ATG5^-/-^ cells (24%) (Fig 5A and 5B). This is in contrast to p62, which labels the parasite PVM in less than 25% of infected LC3B-knockout cells and in less than 1% of ATG5-knockout cells (Fig 4B). This indicates that GFP-NBR1 is able to interact with other proteins, most likely of the ATG8 family or with parasite proteins in the PVM. The distribution of GFP-NBR1 and p62 differs markedly. In both non-infected and infected cells GFP-NBR1 shows a more punctate pattern in contrast to p62, which is more evenly distributed (Figs 4A and 5A). Also the localisation of GFP-NBR1 to the PVM appears more in clusters compared to p62, which is largely evenly distributed over the surface of the PVM. When RFP-LC3 and GFP-NBR1 are simultaneously transfected into HeLa WT and LC3B-deficient HeLa cells, at least 98% of the parasites show a GFP-NBR1-positive PVM with a strong co-localization of both molecules (Fig 5C). This result provides strong evidence that LC3B is indeed able to recruit GFP-NBR1 to the PVM in *P*. *berghei*-infected host cells. It now remains to be shown whether or not GFP-NBR1 is directly recruited by LC3B or through an interaction with p62.

To exclude that we observed indirect binding of RFP-LC3B to GFP-NBR1 mediated through a RFP-GFP interaction, we carried out a control experiment. HeLa WT cells were transfected simultaneously with RFP-LC3B and GFP alone. Approximately 24 hours after transfection, these cells were infected with *Pb*mCherry. Cells were fixed 6 hours post-infection and analyzed by fluorescence microscopy. No interaction between RFP-LC3B and GFP was detected (S3 Fig) confirming that LC3B can indeed specifically recruit the receptor protein NBR1.

### LC3B recruits ubiquitin to the PVM in *P*. *berghei*-infected cells

The PVM of *P*. *berghei* parasites is immediately labeled with the autophagy marker proteins LC3B, p62 and NBR1 after sporozoite infection. As our experiments strongly suggest that p62 and NBR1 labeling is dependent on L3CB, the observed mechanism is obviously different from classical selective autophagy. To determine whether in *P*. *berghei*-infected cells a completely inverted recruitment of autophagy proteins occurs, we next investigated the recruitment of ubiquitin to the PVM in the three different cell lines. GFP-ubiquitin was ectopically expressed in HeLa WT, LC3B- and ATG5-deficient cells and transfected cells were infected with *Pb*mCherry. Six hours post-infection, cells were stained with antibodies and the localization of ubiquitin was monitored visually by fluorescence microscopy.

**Fig 6:**
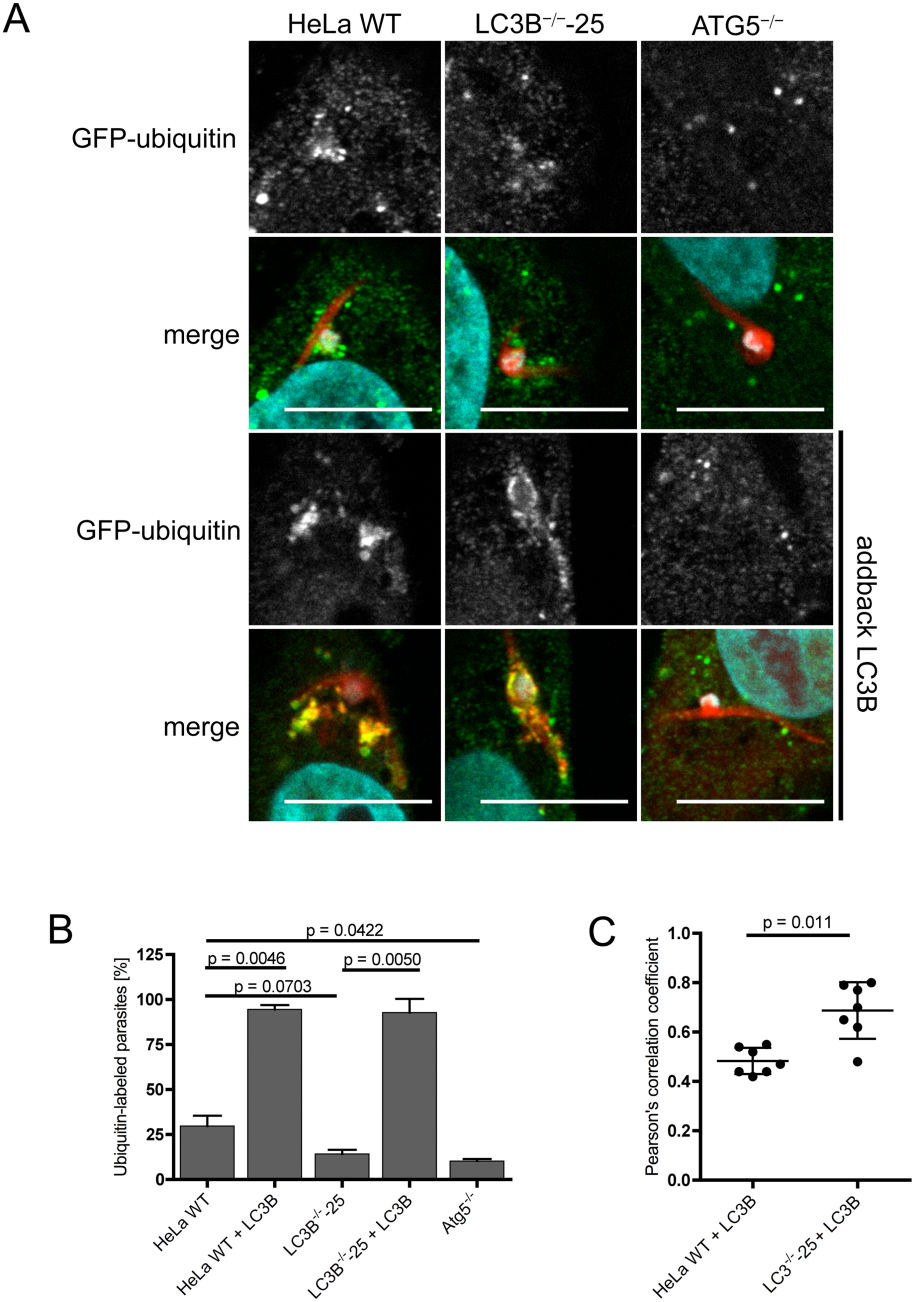
LC3B recruits ubiquitin to the PVM. **(A)** HeLa WT, HeLa LC3B- and ATG5-knockout cells were infected with *Pb*mCherry (red). 6 hours post-infection, cells were fixed and stained with anti-ubiquitin antibodies (green) as a control. Control and knockout cell lines were transfected with RFP-LC3B (two lower panels) and infected 17 hours after transfection with *P*. *berghei* sporozoites expressing mCherry (red). Ubiquitin (green) was visualised using anti-ubiquitin antibodies. DNA was labeled with DAPI (blue). Cells were analyzed by confocal microscopy. Scale bar 10 μm. **(B)** Numbers of ubiquitin-labeled *P*. *berghei* parasites in non-transfected and in RFP-LC3B-transfected cells were determined by fluorescence microscopy. 77–103 parasites were analysed in the non-transfected HeLa cells and 53–83 parasites were analysed for the RFP-LC3B-transfected HeLa cells. Two individual experiments were carried out. Labeled parasites are expressed as percentages. Standard deviations are depicted. **(C)** Pearson’s correlation coefficients of ubiquitin and RFP-LC3B were calculated from seven individual *P*. *berghei* parasites in HeLa WT and LC3B^-/-^ cells transfected with RFP-LC3B. Mean values are 0.4829 (HeLa WT) and 0.6871 (LC3B^-/-^). Depicted are standard deviations.

Ubiquitin-labeled parasites could be detected in HeLa WT, LC3B^-/-^ and in ATG5^-/-^ cells but labeling was strongest in HeLa WT cells (Fig 6A and 6B). In LC3B-deficient cells we observed less ubiquitin-labeled parasites than in WT cells. However, ubiquitin recruitment to the PVM labeling was weakest in ATG5-deficient cells. When HeLa WT and LC3B-deficient cells are transfected with RFP-LC3B, the parasite PVM shows a much stronger labeling with ubiquitin (Fig 6A and 6B). Transfection of ATG5-deficient cells with RFP-LC3B did not show this effect, confirming that the observed phenotype is highly specific (Fig 6A and 6B). These observations further support the notion that LC3B can recruit ubiquitin to the PVM of *P*. *berghei*-infected cells, probably via p62 and NBR1.

## Discussion

Selective autophagy regulates the degradation of specific cellular components and invading pathogens in autophagosomes. Classically, cargo to be degraded becomes ubiquitinated [40]. Subsequently, ubiquitin binding domain-containing (UBD-containing) receptors recognise the ubiquitinated cargo and mediate, via LIR domains, binding of LC3 family proteins [11]. This initiates the formation of an autophagosomal membrane around the cargo, which is finally degraded by fusion with lysosomes. In contrast to classical selective autophagy targeting intracellular cargo or pathogens, in *P*. *berghei*-infected cells, no formation of an autophagosomal membrane occurs. *Plasmodium* parasites reside in a vacuole that is directly targeted by autophagy marker proteins including autophagy receptors and ubiquitin [5,27]. Since no additional autophagosomal membrane is formed, it is safe to assume that receptor association with the PVM has a completely different function. A hint in this direction came from earlier work [5] showing that *Plasmodium* parasites try to avoid autophagic elimination by clearing the PVM of autophagy markers during parasite development [5]. This phenomenon was not only found for LC3 but also for ubiquitin and p62. Interestingly, at the same time, lysosome association with the PVM was strongly reduced suggesting that the parasite actively tries to avoid progression of autophagy by clearing the PVM of autophagy marker proteins. [42] It is tempting to speculate that the autophagy receptors are involved in PVM clearance but the deciphering of the molecular details of this event is beyond the scope of the present study and is a subject of current investigations.

Selective autophagy relies on an arsenal of receptors that target different cargo for degradation: p62 is known to recognise protein aggregates, bacteria, zymogen particles, midbodies and nucleic acids; NBR1 targets aggregated proteins, peroxisomes and midbodies for degradation; NDP52 labels bacteria, mitochondria and nucleic acids; OPTN is involved in degradation of protein aggregates, bacteria and mitochondria (reviewed in [43]). Autophagy receptors do not have an absolute specialization but often collaborate to target a cargo for degradation. p62 and NBR1 interact with each other in aggrephagy [41], pexophagy [19] and midbody ring degradation [44]. In xenophagy, a process that removes invading bacteria, p62 works together with NDP52 and OPTN [22,23,45]. NDP52 and OPTN locate on common subdomains on the ubiquitinated *Salmonella* bacteria containing vacuole, which raises the question whether these two proteins have similar roles in selective autophagy [22]. Ubiquitinated *Salmonella* bacteria are sensed by at least four autophagy receptors: p62, NDP52, OPTN and NBR1. With the exception of NBR1, they all appear to play a role in restricting growth of bacteria [17,22,23,45]. We have found that shortly after infection the PVM in the vast majority of *P*. *berghei-*infected cells is decorated with the autophagy receptor proteins p62 and NBR1 and to a much lesser extent with NDP52. The mechanism of their recruitment appears, however, to be completely different compared to that seen in bacterial infections.

We show that in contrast to our expectations, p62 and NBR1 binding to the PVM depends on LC3B. When we delete LC3B from HeLa cells, PVM labeling by p62 and NBR1 is diminished. Importantly, complementation of the LC3B knockout cell lines with RFP-LC3 fully restores PVM labeling by p62 and NBR1, confirming the central role of LC3B in this process in *P*. *berghei*-infected cells. Another important observation of this work is that depletion of LC3B does not have an obvious effect on autophagosome formation in general, confirming recent work in hexa knock out cells (HeLa cells knocked out for all LC3s and GABARAPs). In these cells, autophagosomes can still be formed and a certain level of autophagy is still possible [39]. We also did not observe an obvious effect on parasite growth in LC3B-negative HeLa cells (data not shown). It might well be that other members of the LC3 protein family partly compensate for the lack of LC3B. This is supported by the notion that in *P*. *berghei*-infected, LC3B-deficient HeLa cells, p62 and even more NBR1 association with the PVM was not completely abolished. However, when we deleted ATG5, which acts upstream in the autophagy cascade, the PVM of *P*. *berghei*-infected cells was never labeled with p62 but moderately labeled with NBR1. This indicates that ATG5-mediated lipidation and incorporation of LC3B or another member of the LC3 family into the PVM is necessary to recruit p62 and NBR1 and that NBR1 might be able to directly bind to other PVM proteins. Interestingly, ubiquitin recruitment to the PVM appears also to be dependent on LC3B presence in this membrane. This became most obvious when RFP-LC3B was co-transfected with GFP-ubiquitin. We reported earlier that in infected HepG2 cells a higher percentage of parasites exhibit ubiquitin labeling of the PVM [5]. It is possible that HepG2 cells express higher levels of LC3 than HeLa cells used in the present study. Together, our experiments show that in *P*. *berghei*-infected cells, the PVM labeling with autophagy proteins is reverted, with LC3B recruiting autophagy receptors and ubiquitin.

So far the role of receptor recruitment to the PVM has not been clear and one can only speculate on its function. For example, it might have a role in signaling pathways that promote survival of the host cell during parasite development. It has been shown that p62 can specifically interact with active RagC/D heterodimers, Raptor and TRAF6 and is able to activate the pro-survival factor mTORC1 on the lysosomal surface [46,47]. p62 can also activate the Keap1-Nrf2 pathway, which is a defense mechanism against oxidative and electrophilic stress [46]. Under normal conditions, Nrf2 is bound to the E3 ubiquitin ligase adaptor protein Keap1, which ubiquitinates Nrf2 and leads to its proteasomal degradation. When autophagic flux is compromised and p62 accumulates, Keap1 is sequestered by p62 and can no longer bind Nrf2, leading to increased Nrf2 signaling and down-regulation of oxidative stress. Since oxidative stress can be employed as a defense strategy against intracellular pathogens, activation of the Nrf2 pathway would support parasite survival. In fact, it has recently been shown that *P*. *berghei*-infected host cells do not show enhanced oxidative stress conditions [48]. It will now be interesting to investigate whether or not there is indeed a link between autophagy receptor signaling and oxidative stress regulation in *P*. *berghei*-infected hepatocytes.

Here we show that in the vast majority of *P*. *berghei*-infected cells, p62 and NBR1 are recruited to the PVM and that this clearly depends on the presence of LC3B. It will now be highly interesting but also very challenging to elucidate the function of this prominent interaction.

## Supporting information

S1 Fig LC3B knockout cells are able to undergo canonical autophagy. **(A)** Representative western blot of non-infected HeLa WT, ATG5-knockout cells and two clonal LC3B-knockout cell lines left untreated or simultaneously treated with 10 μM chloroquine and 250 ng/ml rapamycin for 4 hours. **(B)** HeLa WT and HeLa LC3B knockout cells ectopically expressing GFP-Gate16 were left untreated or treated with 10 μM chloroquine and 250 ng/ml rapamycin for 4 hours. Fixed cells were stained with anti-GFP antibodies to visualise Gate16 (green) or anti-LC3B antibodies (red). DNA was stained with DAPI (blue). White arrows in the LC3B panel indicate Gate16-transfected cells. Yellow arrowheads in the enlarged pictures indicate autophagic structures where Gate16 and LC3B colocalise. Scale bar 20 μm.
(TIF)

S2 Fig LC3B recruits p62 to the PVM. **(A)** HeLa WT, HeLa LC3B- and ATG5-knockout cells were infected with *Pbm*Cherry (red). 6 hours post-infection, cells were fixed and stained with anti-p62 antibodies (green). All cell lines were transfected with RFP-LC3B (two lowest panels) and infected 17 hours after transfection with *P*. *berghei* sporozoites expressing mCherry (red). RFP-LC3B (red) and p62 (green) were visualised using antibodies. DNA was labeled with DAPI (blue). Cells were analysed by confocal microscopy. Scale bar 10 μm. **(B)** Numbers of p62-labeled *P*. *berghei* parasites in non-transfected and in RFP-LC3B-transfected cells were determined by fluorescence microscopy. 100–130 parasites were analysed in the non-transfected HeLa cells and 60–120 parasites were analysed for the RFP-LC3B-transfected HeLa cells. Two individual experiments were carried out. Labeled parasites are expressed as percentages. In the non-transfected cells, the two LC3B- and ATG5-knockout cell lines show significant less p62 associated with the parasite. In RFP-LC3B-transfected knockout cell lines, p62 association is not different to in RFP-LC3B-transfected WT cells. Standard Deviations are depicted.
(TIF)

S3 Fig RFP-LC3B does not recruit GFP. HeLa WT cells were simultaneously transfected with RFP-LC3B and GFP alone. Approximately 24 hours post transfection cells were infected with *Pbm*Cherry and 6 hours post infection cells were fixed and analysed by fluorescence microscopy. In the left panel RFP-LC3B and *Pbm*Cherry are shown. A white arrowhead points towards an LC3B-labeled parasite. The middle panel shows the GFP signal in greyscale. Scale bar 20 μm
(TIF)

S1 Table Sequence analysis of three different LC3B^-/-^ clonal cell lines. Edited regions were amplified by PCR with the three different primer pairs indicated in the table. PCR products were cloned into a plasmid and sequence analysis of 24 individual plasmids was performed for each of the three LC3B knock out cell lines. 1 to 3 different alleles were found in each cell line.
(DOCX)
