# Supplementary figures and images for: Inverted recruitment of autophagy proteins to the Plasmodium berghei parasitophorous vacuole membrane
Source: PLoS One. 2017 Aug 25;12(8):e0183797. doi: 10.1371/journal.pone.0183797 (PMC5571950; doi:10.1371/journal.pone.0183797)

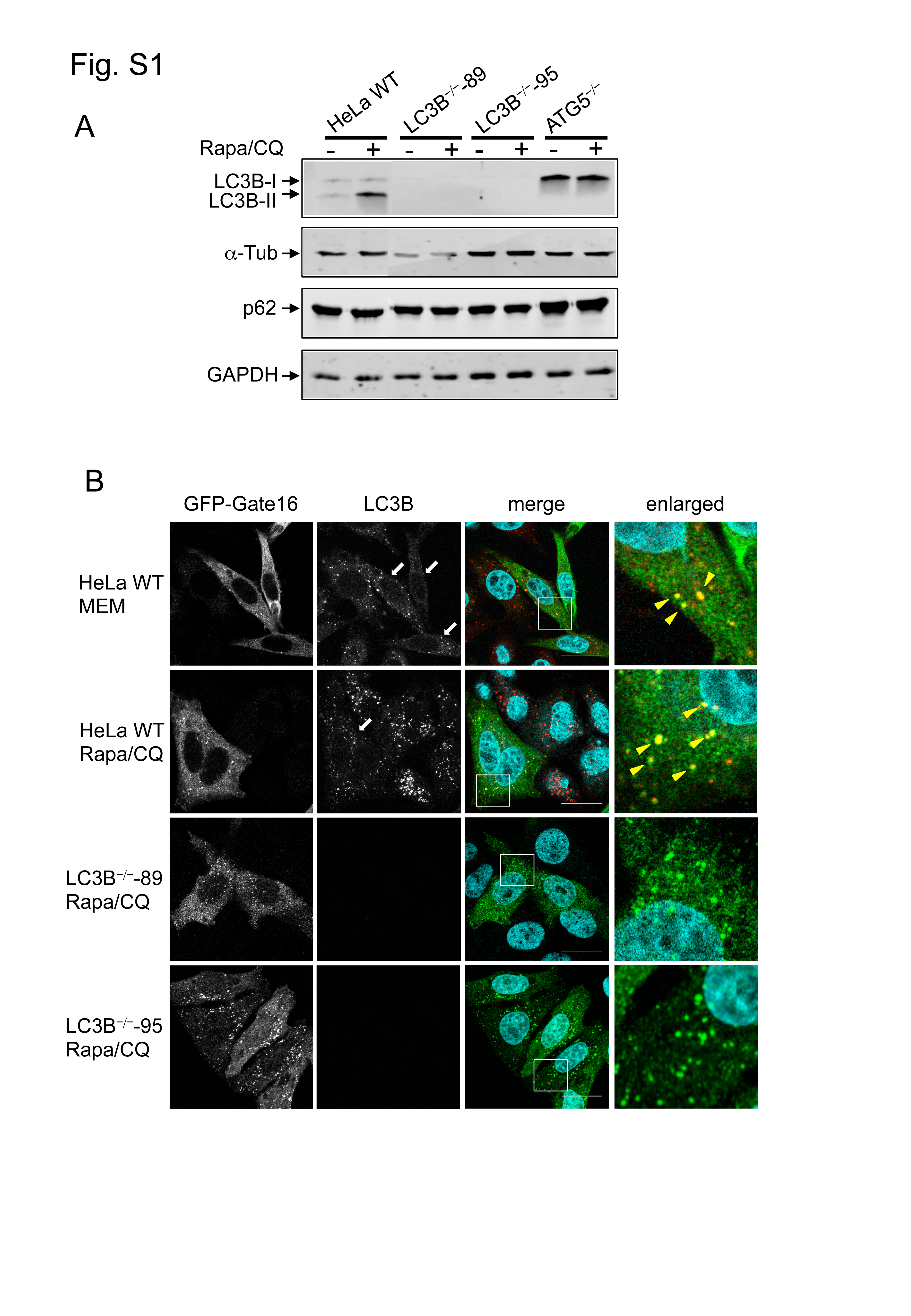

Supplement: S1 Fig — (A) Representative western blot of non-infected HeLa WT, ATG5-knockout cells and two clonal LC3B-knockout cell lines left untreated or simultaneously treated with 10 μM chloroquine and 250 ng/ml rapamycin for 4 hours. (B) HeLa WT and HeLa LC3B knockout cells ectopically expressing GFP-Gate16 were left untreated or treated with 10 μM chloroquine and 250 ng/ml rapamycin for 4 hours. Fixed cells were stained with anti-GFP antibodies to visualise Gate16 (green) or anti-LC3B antibodies (red). DNA was stained with DAPI (blue). White arrows in the LC3B panel indicate Gate16-transfected cells. Yellow arrowheads in the enlarged pictures indicate autophagic structures where Gate16 and LC3B colocalise. Scale bar 20 μm. (TIF) [file pone.0183797.s001.tif]

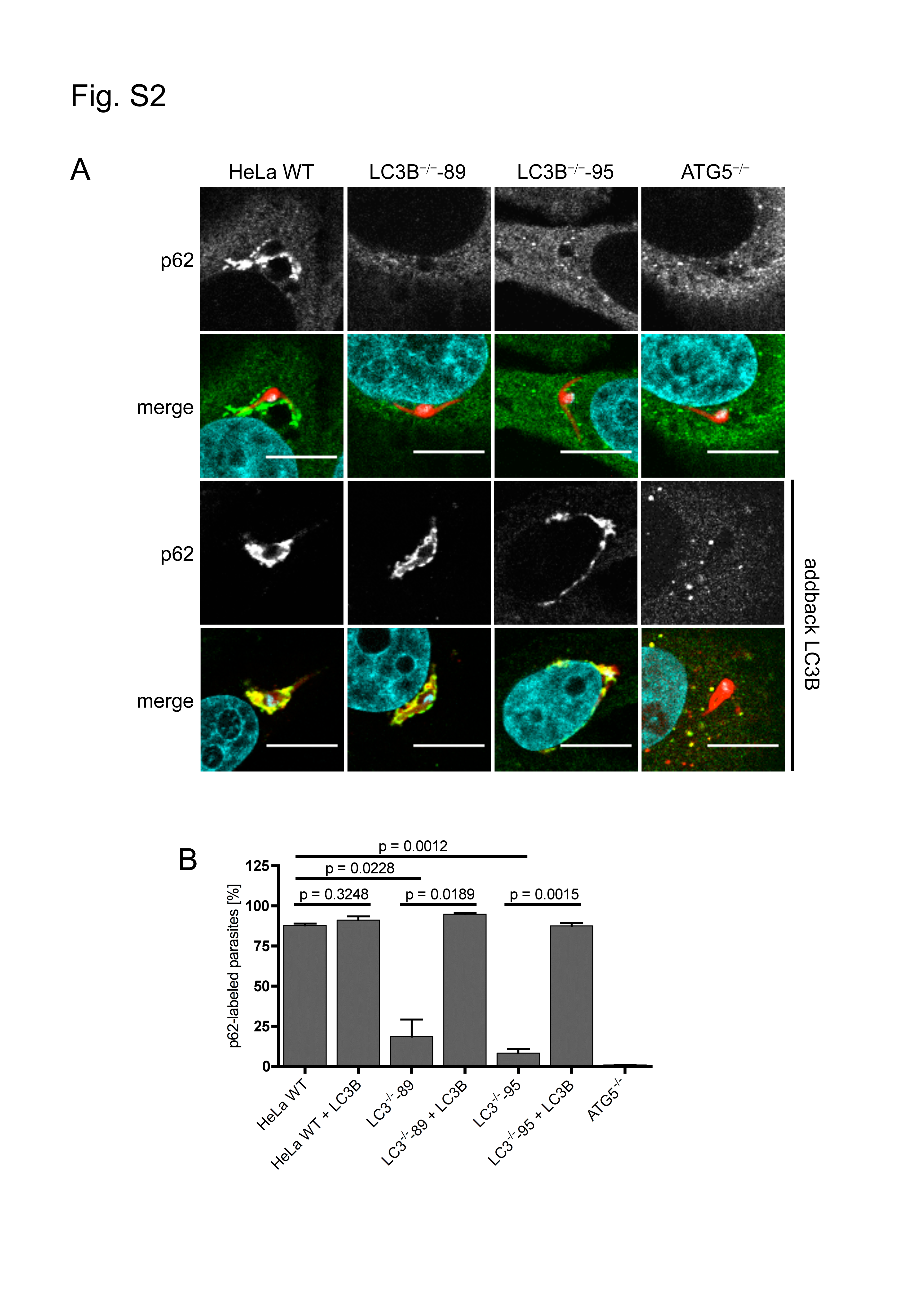

Supplement: S2 Fig — (A) HeLa WT, HeLa LC3B- and ATG5-knockout cells were infected with PbmCherry (red). 6 hours post-infection, cells were fixed and stained with anti-p62 antibodies (green). All cell lines were transfected with RFP-LC3B (two lowest panels) and infected 17 hours after transfection with P. berghei sporozoites expressing mCherry (red). RFP-LC3B (red) and p62 (green) were visualised using antibodies. DNA was labeled with DAPI (blue). Cells were analysed by confocal microscopy. Scale bar 10 μm. (B) Numbers of p62-labeled P. berghei parasites in non-transfected and in RFP-LC3B-transfected cells were determined by fluorescence microscopy. 100–130 parasites were analysed in the non-transfected HeLa cells and 60–120 parasites were analysed for the RFP-LC3B-transfected HeLa cells. Two individual experiments were carried out. Labeled parasites are expressed as percentages. In the non-transfected cells, the two LC3B- and ATG5-knockout cell lines show significant less p62 associated with the parasite. In RFP-LC3B-transfected knockout cell lines, p62 association is not different to in RFP-LC3B-transfected WT cells. Standard Deviations are depicted. (TIF) [file pone.0183797.s002.tif]

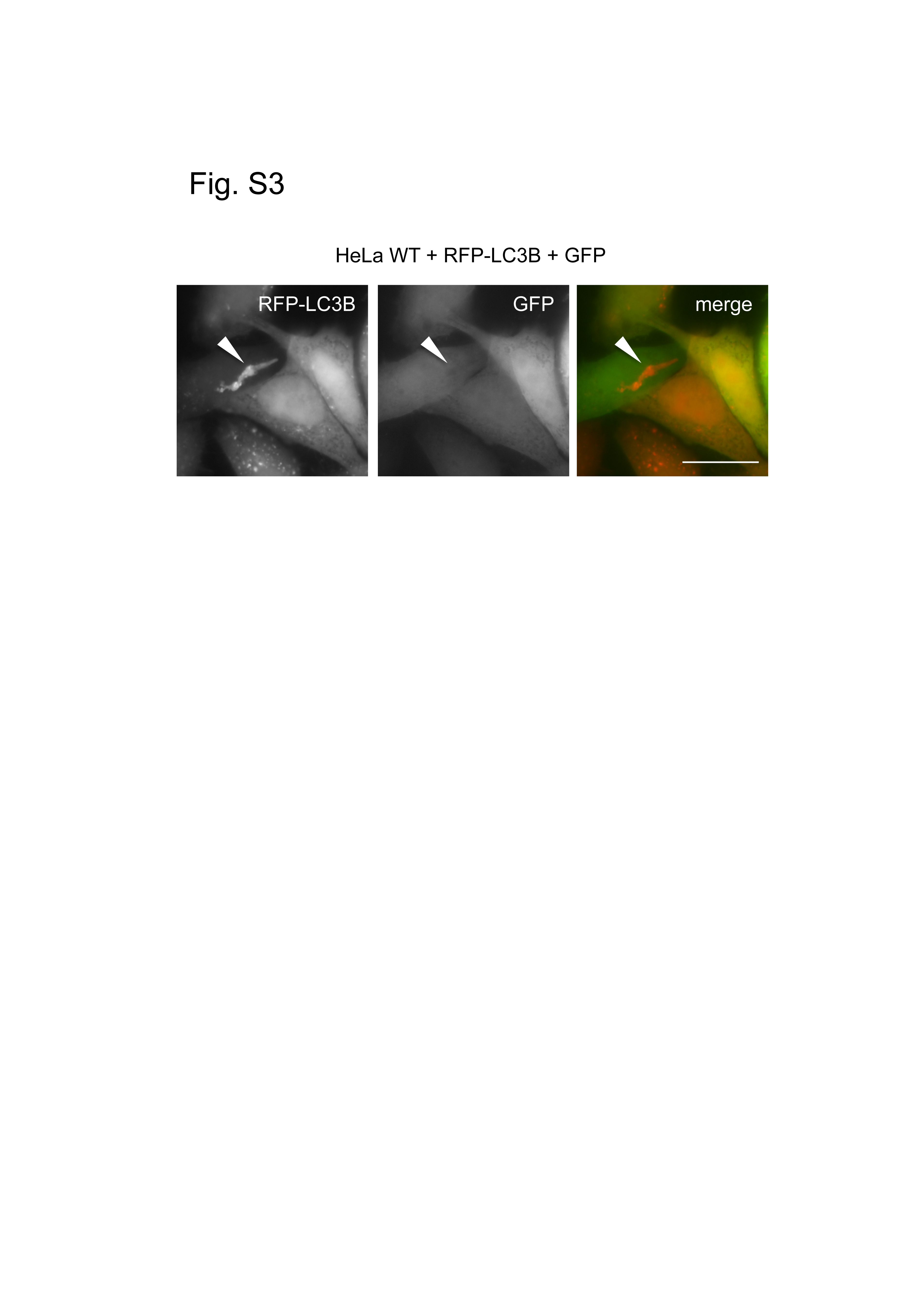

Supplement: S3 Fig — HeLa WT cells were simultaneously transfected with RFP-LC3B and GFP alone. Approximately 24 hours post transfection cells were infected with PbmCherry and 6 hours post infection cells were fixed and analysed by fluorescence microscopy. In the left panel RFP-LC3B and PbmCherry are shown. A white arrowhead points towards an LC3B-labeled parasite. The middle panel shows the GFP signal in greyscale. Scale bar 20 μm (TIF) [file pone.0183797.s003.tif]
