# Supplementary material for: Inverted recruitment of autophagy proteins to the Plasmodium berghei parasitophorous vacuole membrane
Source: PLoS One. 2017 Aug 25;12(8):e0183797. doi: 10.1371/journal.pone.0183797 (PMC5571950; doi:10.1371/journal.pone.0183797)
Supplement: S1 Table — Edited regions were amplified by PCR with the three different primer pairs indicated in the table. PCR products were cloned into a plasmid and sequence analysis of 24 individual plasmids was performed for each of the three LC3B knock out cell lines. 1 to 3 different alleles were found in each cell line. (DOCX) [file pone.0183797.s004.docx]

**S1 Table**: **Genotyping results of LC3B knockout cell lines.**

| **Clone name** | **Genotyping primers** | **PCR product (bp)** | **Editing result** | |
| --- | --- | --- | --- | --- |
| LC3B^-/-^-25 | ACCAAATATCGCATGGTGGT  AACGTAATCGCAAACCCAAG | 1762 | 1 | 13 bp insertion in 5‘-UTR;  454 bp deletion containing a  antiparallel duplication |
| LC3B^-/-^-89 | GTCACCTGACCAGGCTGCGG  GAGGGCGCGGGGTGATTCAG | 508 | 1  2  3 | T-C Transition + T insertion  22 bp deletion  25 bp deletion |
| LC3B^-/-^-95 | CCCACAACCGTCACCTCAG  CCAGAAGCGCGACCCTCG | 798 | 1  2 | 216 bp deletion  22 bp deletion |
